# Development and Characterization of an Effective Food Allergy Model in Brown Norway Rats

**DOI:** 10.1371/journal.pone.0125314

**Published:** 2015-04-29

**Authors:** Mar Abril-Gil, Alba Garcia-Just, Francisco J. Pérez-Cano, Àngels Franch, Margarida Castell

**Affiliations:** 1 Departament de Fisiologia, Facultat de Farmàcia, Universitat de Barcelona, Barcelona, Spain; 2 Institut de Recerca en Nutrició i Seguretat Alimentària, Universitat de Barcelona (INSA-UB), Barcelona, Spain; Université Paris Descartes, FRANCE

## Abstract

**Background:**

Food allergy (FA) is an adverse health effect produced by the exposure to a given food. Currently, there is no optimal animal model of FA for the screening of immunotherapies or for testing the allergenicity of new foods.

**Objective:**

The aim of the present study was to develop an effective and rapid model of FA in Brown Norway rats. In order to establish biomarkers of FA in rat, we compared the immune response and the anaphylactic shock obtained in this model with those achieved with only intraperitoneal immunization.

**Methods:**

Rats received an intraperitoneal injection of ovalbumin (OVA) with alum and toxin from *Bordetella pertussis*, and 14 days later, OVA by oral route daily for three weeks (FA group). A group of rats receiving only the i.p. injection (IP group) were also tested. Serum anti-OVA IgE, IgG1, IgG2a, IgG2b and IgA antibodies were quantified throughout the study. After an oral challenge, body temperature, intestinal permeability, motor activity, and mast cell protease II (RMCP-II) levels were determined. At the end of the study, anti-OVA intestinal IgA, spleen cytokine production, lymphocyte composition of Peyer’s patches and mesenteric lymph nodes, and gene expression in the small intestine were quantified.

**Results:**

Serum OVA-specific IgG1, IgG2a and IgG2b concentrations rose with the i.p. immunization but were highly augmented after the oral OVA administration. Anti-OVA IgE increased twofold during the first week of oral OVA gavage. The anaphylaxis in both IP and FA groups decreased body temperature and motor activity, whereas intestinal permeability increased. Interestingly, the FA group showed a much higher RMCP II serum protein and intestinal mRNA expression.

**Conclusions:**

These results show both an effective and relatively rapid model of FA assessed by means of specific antibody titres and the high production of RMCP-II and its intestinal gene expression.

## Introduction

Food allergy (FA) is ‘an adverse health effect arising from a specific immune response that occurs reproducibly on exposure to a given food’ [1]. Nowadays it is a major public health problem and the only therapy available consists of avoiding the causative foods [2]. An American retrospective study showed that the economic burden of FA reactions and anaphylaxis treatments is near to $300 million [3]. Despite the fact that more than 170 foods have been reported to cause IgE-mediated hypersensitivity [4], most of the allergic reactions are attributed to a limited number of foods, cow’s milk, egg, nuts and seafood being the most common in Europe [5], whereas they share prominence with wheat, soy and peanut in the USA [6]. Although the exact prevalence of FA remains uncertain, data supports that its prevalence is increasing with current rates around 5% in adults and approaching 8% in the child population [7].

In healthy conditions, the intestinal barrier, constituted by the epithelium covered with mucus, enzymes and bile salts together with extreme pH, acts as a physical barrier preventing the passage of harmful pathogens, as well as a selective filter, allowing essential dietary nutrients to pass into the circulation [8,9]. In general, food ingestion results in oral tolerance: when dendritic cells, the professional antigen-presenting cells, capture food antigen in the lamina propria (LP) and Peyer’s patches (PP), they carry them to the mesenteric lymph nodes (MLN) where they induce regulatory T (Treg) cells that migrate back to the LP. The resident macrophages in the LP can expand Treg cells, suppressing Th2 cytokines and IgE as well as the effector functions of mast cells and basophils, thus inhibiting allergic inflammation and food hypersensitivity [8,10]. In contrast, patients with FA have lost the immune mechanisms responsible for oral tolerance, and recognize some food antigens as harmful molecules. In this population, alterations in Treg cell function and environmental factors, such as microbiota, have been suggested to be important contributors to food sensitization and allergy [11].

Animal models, such as those described in dogs, swine, guinea pigs, mice and rats, have been used for assessment of allergenicity of foods, although the optimal model has not been reached [12–18]. In the case of dogs, the gut anatomy, physiology and nutritional requirements are similar to humans and in swine the anatomy, physiology and immunology of skin and gastrointestinal tract are also comparable to humans [19], but in both animal species there are some disadvantages in comparison with rodents, such as the expense incurred by animal maintenance, the limited availability of strains, the lack of commercially available immunological reagents, and the long process to sensitization (18 months for dogs) [20]. Studies related to cow’s milk allergy commonly use guinea pigs for oral sensitization [21,22]. However, it is not an appropriate model for the assessment of allergenicity of novel proteins because the immunological reactions to proteins differ from those in humans [22], there are a lack of available tools to study the guinea pig immune system and, for FA research, there are significant differences in the immunophysiology in comparison with other species [19].

Regarding the use of mice in allergy research, the transcriptional analysis approach has shown remarkable consistency between murine and human samples, and studies in atopic dermatitis showed a high degree of homology in the gene expression profile [23]. In addition, their small size, short breeding cycle and well-characterized immunology are certainly key factors. Several allergy models performed in mice differ in the strain, the sensitization route, the type of allergen, the dosage, or the use of an adjuvant [16,24–27]. Nevertheless, the natural complexity of the allergic reactions makes it difficult to find a single reliable marker to quantify the sensitization potential of a protein [28]. Finally, the use of rats has a number of advantages compared with other animal models, particularly with respect to being one of the most commonly used species in toxicity testing [29]. Brown Norway (BN) rats have been widely studied because this strain is a high IgE responder, similar to atopic humans. BN rats have been used as a model of FA in the presence or absence of an adjuvant. In this latter condition, Knippels et al. have demonstrated oral sensitization and have evaluated the influence of rat strain [30] and dosage [31,32]. However, the model of oral sensitization without an adjuvant requires a long process of sensitization (six weeks) and, although it has been used in several studies [33–36], success after oral sensitization was not always achieved in a high percentage of rats [37] and/or the sensitization does not always induce the synthesis of IgE antibodies [20,30,38,39]]. This limitation makes it difficult to use this model for the screening of new therapies or allergenicity studies. Regarding the use of other sensitization routes and an adjuvant to induce FA in BN rats, the administration of two to three intraperitoneal (i.p.) injections of allergen and, in some cases, the oral gavage of the same allergen has been applied [40–42]. The present study aimed to develop an effective and more rapid model of FA in BN rats based on that reported by Ogawa et al. [43] with only one i.p. injection of the allergen with alum together with toxin from *Bordetella pertussis* (tBp) to promote IgE synthesis [44], and two weeks later the oral administration of soluble allergen. In order to establish biomarkers of FA in rat, we compared the specific immune and the anaphylactic responses obtained in this model with those achieved with only an i.p. immunization.

## Material and Methods

### Animals and experimental design

Three-week-old female BN rats obtained from Janvier (Saint-Berthevin, France) were maintained on an OVA-free diet and water *ad libitum*. The parent rats had followed the SSNIFF S8189-S105 diet, free of egg proteins. The rats were housed in cages under conditions of controlled temperature and humidity in a 12:12 h light-dark cycle. After an acclimatization period of one week, the rats were randomized into three groups: reference (RF) group, intraperitoneal (IP) group and food allergy (FA) group (n = 8 per group). The FA induction was carried out by combining an i.p. immunization with OVA mixed with alum and tBp followed, 14 days later, by oral OVA administration for three weeks; five days later, an oral challenge was given to cause an anaphylactic response (AR). The AR was evaluated by means of body temperature, protease release of mast cells, intestinal permeability and also by motor activity assessment [45]. Finally, rats were sacrificed on day 42, two days after the oral challenge, to collect tissue samples. During the study, the body weight was registered and blood samples were collected weekly to determine specific antibodies production.

Experimental design was repeated twice in order to get representative results of an enough number of animals per group.

Experimental procedures in rats were reviewed and approved by the Ethical Committee for Animal Experimentation at the University of Barcelona (ref.359/12).

### Food allergy induction

An emulsion of OVA (grade V, Sigma-Aldrich, Madrid, Spain) as allergen, in alum (Imject, Pierce, IL, USA) as an adjuvant and tBp (Sigma-Aldrich) was prepared. Each rat from the IP group received by i.p. route 0.5 mL of the emulsion containing 50 μg of OVA, 2.5 mg of Imject and 50 ng of tBp. In the FA group, in addition to the i.p. injection as administered in the case of the IP group, the animals received, starting 14 days later, 1 mL of OVA solution in sodium bicarbonate (1 mg per rat) by oral gavage five days/week for three weeks. As a control, the IP and RF groups received 1 mL of sodium bicarbonate by oral gavage for the same period.

### Anaphylaxis induction

Forty days after OVA i.p. immunization, the animals were deprived of food overnight and then received 2 mL of OVA (200 mg per rat) orally. Blood was collected every 30 min up to 2 h post-AR induction from the saphenous vein. During this period rectal temperature was measured using a digital thermometer (OMRON Healthcare Europe, the Netherlands).

In order to determine the intestinal barrier integrity, 30 min after the challenge each rat received 100 mg/mL of β-lactoglobuline (βLG, Sigma-Aldrich) by oral gavage [31], details are described in the “Quantification of intestinal permeability” section

### Motor activity measurement

Motor activity was assessed for 21 min using individual cages in an isolated room, with an activity meter that included two perpendicular infrared beams, which crossed the cage 6 cm above the floor. These facilities have been commonly used to study rat motor activity in different conditions [46,47]. Two motor activity measures were performed: the first was measured 24 h before anaphylaxis induction to determine the basal movements, and the second immediately after the oral challenge to establish the changes produced by anaphylaxis induction. Activity counts were recorded using time frames of 1 min for 21 min. To stimulate rat movements, 8 min after the beginning of the measurement, the lights were turned off for 5 min and then turned on until the end of the measurement. The results refer to the movements in three time phases (pre-darkness, darkness and post-darkness) as well as the entire period. The area under the curve (AUC) for the 21-min period and the percentage of decrease in motor activity after AS induction with respect to the basal measurement in each studied phase as well as in the whole period were also calculated.

### Sacrifice and sample processing

Two days after AR the rats were anaesthetized with ketamine (90 mg/kg) (Merial Laboratories S.A, Barcelona, Spain) and xylazine (10 mg/kg) (Bayer A.G, Leverkusen, Germany). Blood was obtained by heart puncture. MLN and spleen were also dissected for immediate lymphocyte isolation. From the middle of the small intestine (SI), a small piece (0.5 cm) was excised and kept in RNA later (Ambion, Life Technologies, Austin, USA) until gene expression analysis by real-time PCR, the procedure is detailed in the “Quantification of gene expression in small intestine” section. From the distal part of the SI, visible PP were collected for immediate lymphocyte isolation, and gut washes were obtained for quantification of specific IgA.

### Peyer’s patches lymphocyte isolation and gut wash obtention

The processing of these samples was performed as previously described [48,49]. Briefly, PP were incubated with complete culture medium containing Roswell Park Memorial Institute (RPMI 1640, Sigma-Aldrich), 10% fetal bovine serum (FBS), 100 IU/mL streptomycin-penicillin, 2 mM L-glutamine (Sigma-Aldrich), and 0.05 mM 2-β-mercaptoethanol (Merck, Darmstadt, Germany) with 1 mM of dithiothreitol (Sigma-Aldrich) (5 min, 37°C). Thereafter, PP were washed with RPMI medium and passed through a cell strainer (40 μm, BD Biosciences, Madrid, Spain).

The remaining distal SI tissue (without PP) was cut into 5 mm pieces, weighed and used to obtain the gut wash by shaking in phosphate-buffered saline (PBS) (37°C, 10 min). Gut washes were conserved at -20°C for anti-OVA IgA determination.

### Ovalbumin-specific stimulation of mesenteric lymph nodes and spleen lymphocytes

MLN and spleen cell suspensions were obtained as previously described [48] by passing the tissue through a cell strainer (40 μm, BD Biosciences). Erythrocytes from the spleen were eliminated by osmotic lysis. MLN and spleen cells were cultured at 5 × 10^6^ cells in 1 mL of medium with or without OVA (50 μg/mL) for 96 h. Supernatants from spleen cultures were collected to assess cytokine concentrations. MLN cells were used to establish changes in lymphocyte composition after specific stimulation.

### Assessment of lymphocyte composition in Peyer’s patches and mesenteric lymph nodes

Peyer’s patches and MLN lymphocytes were stained with the following mouse anti-rat monoclonal antibodies (mAb) conjugated to fluorescein isothiocyanate, phycoerythrin or allophycocyanin: anti-TCRαβ (R73), anti-CD4 (OX-35), anti-CD8α (OX-8), anti-CD45RA (OX-33), anti-NKR-P1A (10/78), anti-CD25 (OX-39) (BD Biosciences) and anti-IgA (Abcam, Cambridge, UK). Cells were labeled with saturating concentrations of conjugated mAb in PBS containing 1% FBS and 0.09% Na_3_N as previously described [50]. Negative control staining using isotype-matched mAb was included for each sample.

Analyses were performed using a FC 500 Series Flow Cytometer (Beckman Coulter, FL, USA), and data were assessed by the FlowJo v7.6.5 software (Tree Star Inc,. Ashland, OR, USA). Lymphocyte populations were defined as: B (CD45RA^+^CD4^−^), B expressing IgA (IgA^+^CD45RA^+^), T (TCRαβ^+^), Th (TCRαβ^+^CD4^+^), Tc (TCRαβ^+^CD8^+^) and activated Th (TCRαβ^+^CD4^+^CD25^+^) cells. Results are expressed as percentages of positive cells in the lymphocyte population previously selected according to their forward scatter and side scatter characteristics.

### Quantification of serum mast cell protease II

In serum samples obtained during the AR, rat mast cell protease II (RMCP-II) concentration was quantified using a commercial ELISA set (Moredun Animal Health, Edinburgh, UK) with slight modifications. In brief, 96-well ELISA plates (Nunc Maxisorp, Wiesbaden, Germany) were coated with anti-rat RMCP-II antibody (overnight, 4°C). After blocking and washing, appropriately diluted serum samples were incubated for 3 h. Peroxidase-conjugated anti-rat RMCP-II antibody was incubated for 2 h and, finally, a 3,3’,5,5’-tetramethylbenzidine solution with H_2_O_2_ was added, and optical density (OD) was measured on a microtiter plate photometer (Labsystems Multiskan, Helsinki, Finland). Data were interpolated by means of Ascent v.2.6 software (Thermo Fisher Scientific, S.I.U., Barcelona, Spain).

### Quantification of intestinal permeability

To assess intestinal permeability, a method previously described in BN rats was used [30,51]. In this method, βLG was orally given 30 min after the OVA challenge and then were quantified by ELISA in serum obtained every 30 min duringanaphylaxis. In brief, ELISA plates were coated with rabbit anti-bovine βLG antibody (A10-125A, Bethyl, Montgomery, USA) and incubated overnight at room temperature. The plates were then blocked with bovine serum albumin (Sigma-Aldrich) in TRIS-buffered saline containing 0.05% Tween 20, and after washing, appropriate diluted samples and standard dilutions were added. Finally, an adequate dilution of peroxidase-conjugate anti-bovine βLG antibody (A10-125P, Bethyl) was incubated and an *o*-phenylenediamine dihydrochloride solution was added for detection of βLG from samples. OD was measured as detailed above.

### Determination of cytokines released from spleen lymphocytes

IL-2, IL-4, IL-10 and IFN-γ cytokines released from spleen cell cultures were measured using the BD Cytometric Beads Assay Rat Soluble Protein Flex Set (BD Biosciences). Briefly, samples and standards were incubated with a mix of specific fluorescent beads for each cytokine. Then, a mix containing the detection antibodies conjugated with phycoerythrin was incubated and, after that, samples were washed. Analysis was carried out by a BD FACSAria (BD Biosciences) cytometer and the FCAP Array Software (BD Biosciences). The limits of detection were 0.46 pg/mL for IL-2, 3.4 pg/mL for IL-4, 19.4 pg/mL for IL-10 and 6.8 pg/mL for IFN-γ.

### Quantification of gene expression in small intestine

For RNA isolation, samples from the SI were processed as previously described [52]. Tissue samples were homogenized in a FastPrep (MP Biomedicals, Illkirch, France) for 30 s. Total RNA was isolated with the RNeasy Mini Kit (Qiagen, Madrid, Spain) following the manufacturer’s recommendations. The quality of the RNA was assessed by the Agilent 2100 Bioanalyzer with the RNA 6000 LabChip kit (Agilent Technologies, Madrid, Spain). Two micrograms of total RNA were converted to cDNA using random hexamers (Life Technologies). The specific PCR TaqMan primers and probes (Applied Biosystems, Weiterstadt, Germany) used were: *Iga* (331943, made to order), *Fcer1a* (Rn00562369_m1, inventoried (I)), *Il2* (Rn00587673_m1, I), *Il4* (Rn01456866_m1, I), *Il10* (Rn00563409_m1, I), *Ifng* (Rn00594078_m1, I) and *Mcpt2* (Rn00756479_g1, I). Quantification of the genes of interest was normalized to the endogenous control *Hprt1* (Rn01527840_m1, I). Real-time PCR assays were performed in duplicate using an ABI Prism 7900HT sequence detection system (Applied Biosystems). The SDS software (version 2.4) was used to analyzethe expression data.

The amount of target mRNA relative to HPRT expression and relative to values from the RF group was calculated using the 2^-ΔΔCt^ method, as previously described [53]. Ct is the cycle number at which the fluorescence signal of the PCR product crosses an arbitrary threshold set within the exponential phase of the PCR. Results are expressed considering gene expression in the RF group as 100%.

### Anti-OVA antibody quantification

Anti-OVA IgG1, IgG2a, IgG2b and IgA antibody concentrations were quantified using an indirect ELISA, and OVA-specific IgE concentration by an antibody-capture ELISA as previously described [54]. The relative concentration of each anti-OVA Ig isotype was calculated by comparison with a pool of OVA-immunized rat sera to which arbitrary units (AU) were assigned according to the dilution of the serum samples used for each isotype determination. The AU/mL assigned were 100000 AU/mL for IgG1 and IgG2a, 10000 AU/mL for IgG2b, 50 AU/mL for IgA, and 10 AU/mL for IgE.

### Statistical analysis

The software package IBM SPSS Statistics 20 (SPSS Inc., USA) was used. The Levene and the Kolmogorov-Smirnov tests were applied to assess variance equality and normal distribution, respectively. Two-way ANOVA tests were used to study the effect of group and group x time interaction. The motor activity data were analyzedby two-way ANOVA for repeated measures considering the group (FA *vs*. IP *vs*. RF group) and time as the interacting factor, followed by Bonferroni’s *post hoc* test. To evaluate the correlation among studied variables, Pearson’s coefficient (ρ) was applied. To analyzethe results from anti-OVA antibodies, RMCP-II, βLG and cytokine concentrations, body temperature, relative gene expression, AUC of motor activity, and lymphocyte composition, non-parametric tests (Kruskal–Wallis and Mann–Whitney U) were used due to non-variance homogeneity. Differences were considered statistically significant for *p* values < 0.05.

## Results

### Body weight and mortality

Rats weighed 66.6 ± 3.68 g (mean ± S.E.M.) at the beginning of the study. Rat growth was monitored throughout the study and was similar among groups. At the end of the study, body weight was 137.1 ± 6.88 g [127.2–146.2], 136.7 ± 4.49 g [130.5–141.6], and 138.4 g ± 3.22 g [135.1–143.7] in the RF, IP and FA groups, respectively. No death was produced after the oral challenge in any of the experimental groups.

### Serum and intestinal anti-OVA antibodies

**Fig 1:**
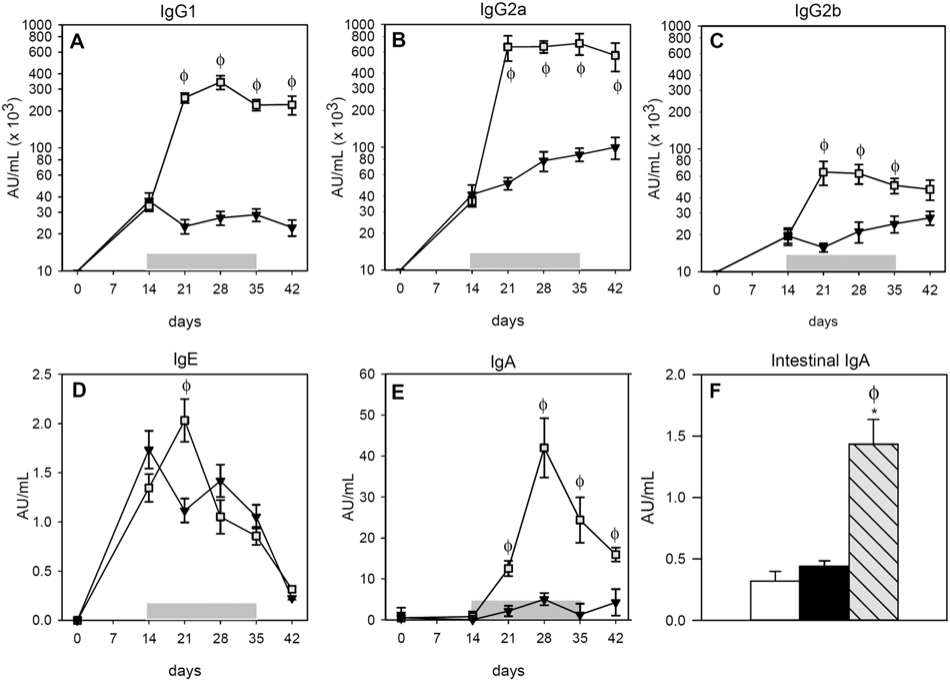
Concentrations of OVA-specific antibodies during post-immunization period. **A)** serum IgG1, **B)** serum IgG2a, **C)** serum IgG2b, **D)** serum IgE, **E)** serum IgA and **F)** intestinal IgA. White bars represent RF group, ▼ or black bars represent IP group and ∎ or grey-striped bars represent FA group. Shadow period corresponds to oral administration of OVA in FA group. Results are expressed as mean ± S.E.M. (n = 8). *p < 0.05 *vs*. RF group and ^ϕ^ *p* < 0.05 *vs*. IP group.

Sera from the RF group did not contain anti-OVA antibodies of any isotype (data not shown). The i.p. immunization caused the synthesis of anti-OVA IgG1, IgG2a and IgG2b antibodies in the IP and FA groups that were already detectable 14 days after OVA immunization (Fig 1A–1C). The oral administration of the allergen boosted the synthesis of anti-OVA IgG isotypes, which increased in the FA group more than tenfold for IgG1 and IgG2a, remaining elevated until the end of the study (Fig 1A and 1B; *p* < 0.05). This increase was also produced in anti-OVA IgG2b, but to a lower degree (Fig 1C; *p* < 0.05).

Regarding serum anti-OVA IgE antibodies (Fig 1D) the OVA immunization also induced their synthesis in both the IP and FA groups. Nevertheless, the oral administration of OVA for a week magnified the production of this antibody in the FA group, increasing almost twofold the levels of specific IgE with respect to the IP group (*p* < 0.05). Afterwards, however, anti-OVA IgE underwent a progressive decrease in both the IP and FA groups.

With regards to the anti-OVA IgA concentrations measured in serum and gut wash samples, the i.p. immunization did not induce the synthesis of this antibody in either compartment (Fig 1E and 1F). In contrast, the oral OVA administration in the FA group induced the synthesis of anti-OVA IgA antibodies (Fig 1E) and they were also found in gut washes at the end of the study (Fig 1F).

### Assessment of anaphylaxis

Body temperature, RMCP-II concentration and intestinal permeability, together motor activity, allowed to quantify anaphylaxis in rats after oral OVA challenge.

**Fig 2:**
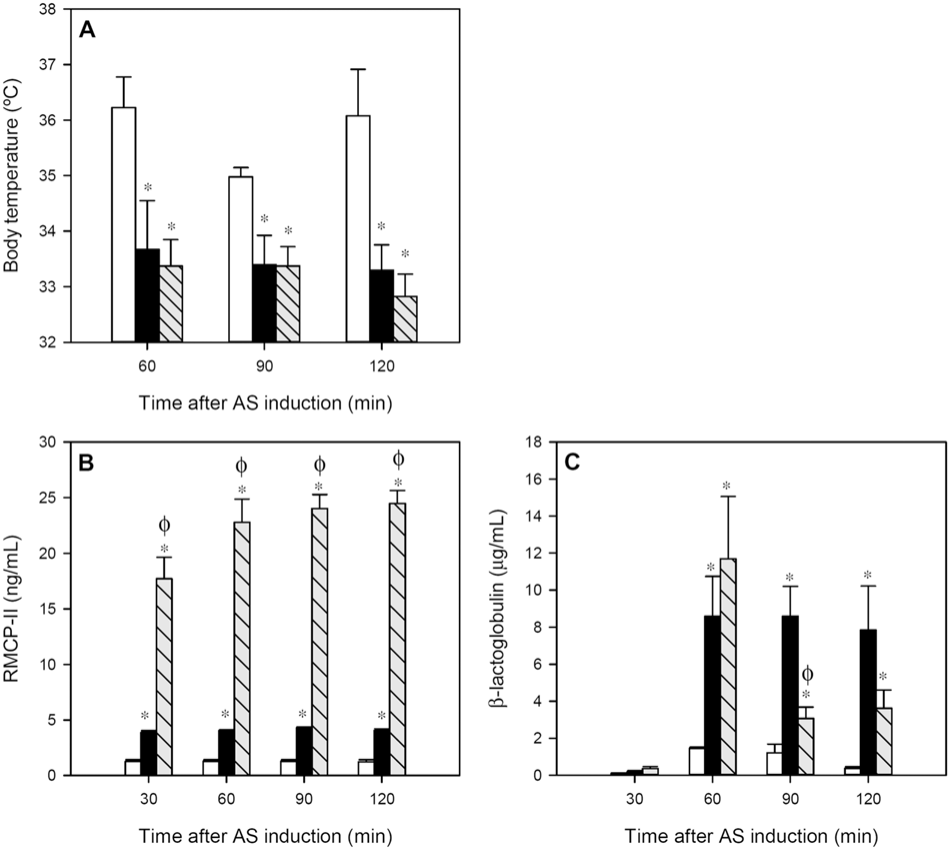
Variables measured during 2 h after anaphylactic shock induction: A) body temperature, B) serum RMCP-II concentration and C) serum βLG concentration. White bars represent RF group, black bars represent IP group and grey-striped bars represent FA group. Results are expressed as mean ± S.E.M. (n = 8). *p < 0.05 *vs*. RF group and ^ϕ^ *p* < 0.05 *vs*. IP group.

The body temperature, registered during the 2 h after oral challenge in intervals of 30 min, revealed that there was a decrease of about 2°C in both the IP and FA groups compared to the RF group throughout the whole studied period (Fig 2A; *p* < 0.05). No significant differences were observed between the IP and FA groups.

After AR induction, the IP group showed about a threefold increase in serum RMCP-II concentration compared to that in RF animals (Fig 2B; *p* < 0.01). However, in the FA group the increase was much higher. The FA animals underwent a rise about 18 times (*p* < 0.01) higher than that of the RF animals and six times higher compared with the IP group (*p* < 0.01). This effect lasted for at least 2 h post-challenge.

βLG given orally 30 min after AR induction, quantified in sera as a measure of intestinal permeability, increased significantly at 30 min from oral protein administration (60 min after AR induction) in both IP and FA groups (Fig 2C; *p* < 0.05). Later, IP rats kept the serum βLG concentration whereas the FA rats showed a faster decrease, although at the end of the studied period, both groups had significantly higher levels compared to RF animals (*p* < 0.05).

### Motor activity

**Fig 3:**
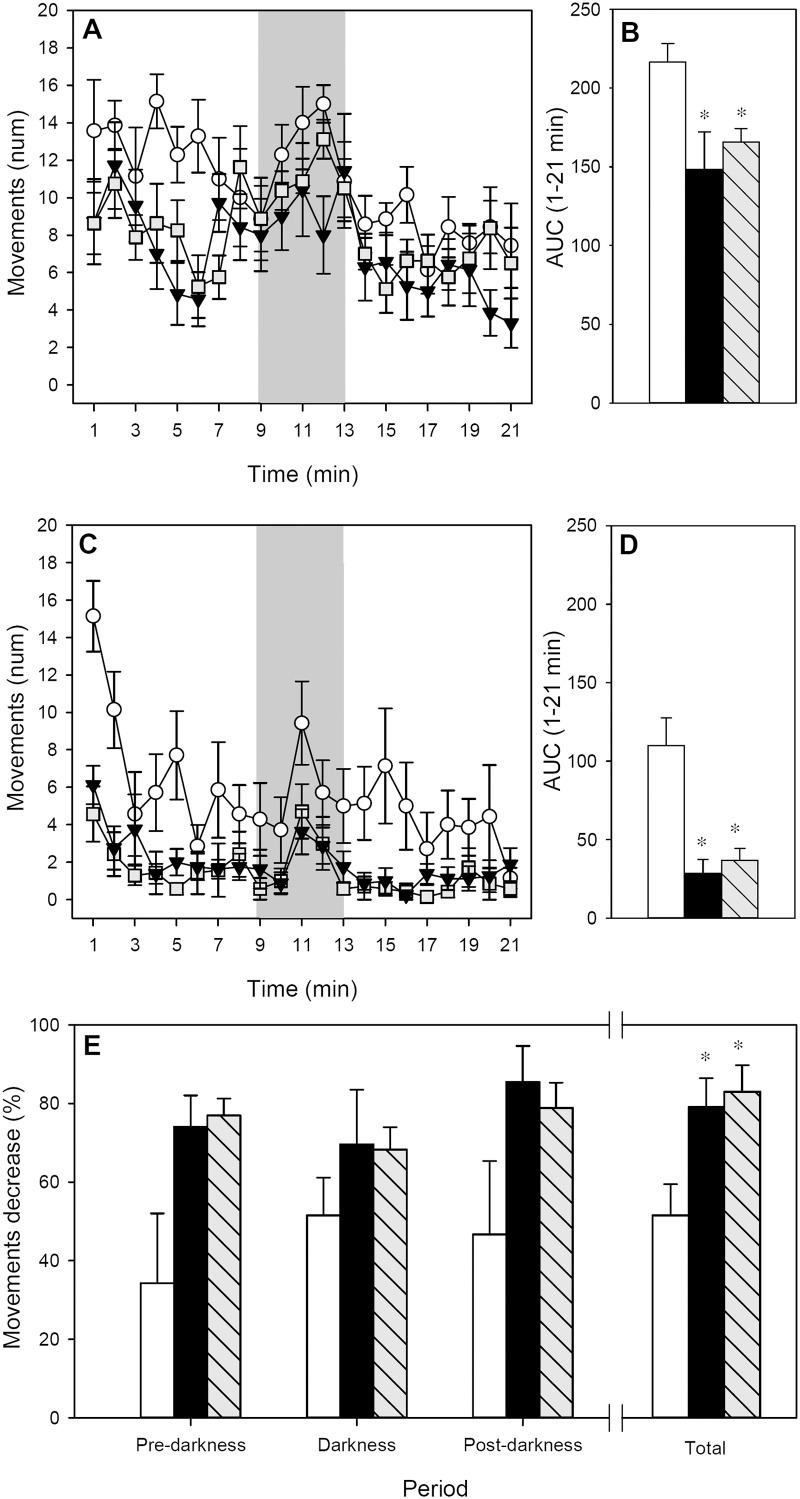
Motor activity for 21-min period. **A)** Basal motor activity assessed 24 h before the AR induction; **B)** area under the curve from the whole studied period before AR induction; **C)** motor activity assessed immediately after AR induction; **D)** area under the curve from the whole studied period after AR induction; **E)** percentage of motor activity decrease after AR induction referring to pre-darkness, darkness, post-darkness and the whole period. ○ or white bars represent RF group, ▼ or black bars represent IP group and ∎ or grey-striped bars represent FA group. In A and C, shadow period corresponds to darkness. Results are expressed as mean ± S.E.M. (n = 8). *p < 0.05 *vs*. RF group.

Rat motor activity was measured for 21 min at 24 h before (Fig 3A) and immediately after (Fig 3C) AR induction to obtain basal values and data representative of AR-induced behavioral changes, respectively. With regards to basal motor activity, the pattern of movements during the time showed that the three groups became quieter over the 21 min period (Fig 3A; *p* < 0.05 for time) although motor activity increased when the lights were turned off (*p* < 0.05 for RF and FA groups). The motor activity of the IP group was lower than that of the RF group, looking at the whole period and the three established phases (pre-darkness, darkness and post-darkness) (*p* < 0.05). Similarly, in the basal pattern, FA rats also made a lower number of movements than RF animals, taking into account the whole period (*p* < 0.001) and also the pre- and post-darkness phases (*p* < 0.05). The differences among basal groups’ movements in the whole studied period can also be observed when AUC was calculated (Fig 3B; *p* < 0.05 IP and FA groups *vs*. RF).

The motor activity registered after AR induction showed a similar pattern to the basal one, the animals being quieter during the pre-darkness phases and more active in the darkness period (Fig 3C; *p* < 0.05). However, the three studied groups showed a lower number of movements than those observed in basal conditions. Interestingly, for those animals belonging to the IP and FA groups, the AR induction produced a more noticeable decrease in the motor activity than in the RF group (*p* < 0.001), which can also be observed when considering the AUC of the whole period (Fig 3D; *p* < 0.05 IP and FA groups *vs*. RF).

The reduction in motor activity resulting from AR induction was also calculated as the percentage of motor activity decrease between basal and post-AR induction in each phase (Fig 3E). RF animals reduced by about 35–50% their number of movements; however, both IP and FA groups underwent a 70–85% reduction of motor activity (*p* < 0.05 in the whole studied period).

There was a correlation between the percentage of decrease in motor activity and the body temperature after AR (ρ = -0.615, *p* < 0.05 at 90 min; ρ = -0.601, *p* < 0.05 at 120 min) meaning that the higher the percentage of decrease, the lower the animal’s body temperature.

### Lymphocyte composition in Peyer’s patches and mesenteric lymph nodes

**Fig 4:**
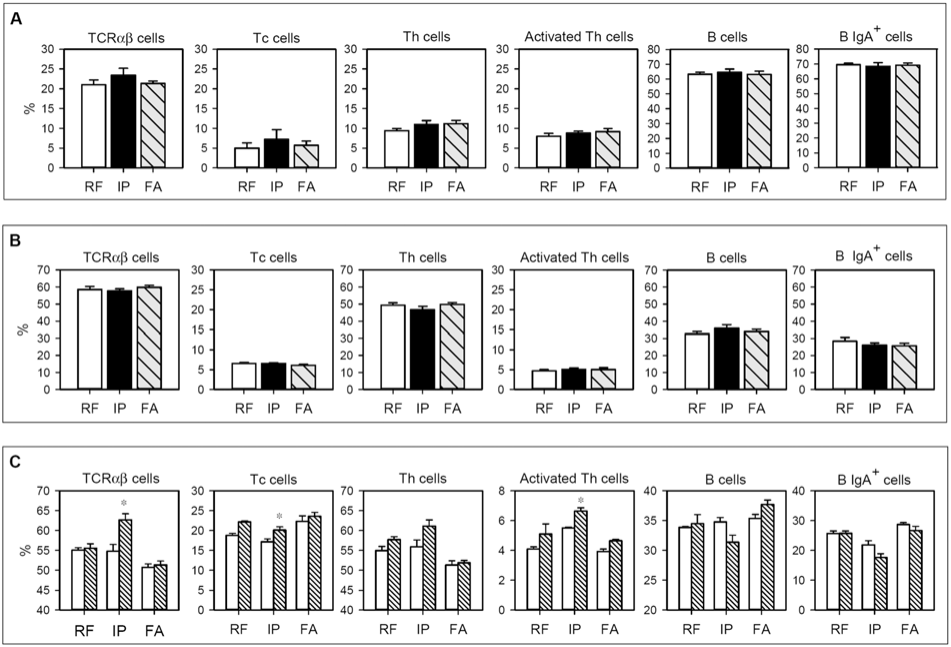
Lymphocyte composition isolated from A) Peyer’s patches, B) mesenteric lymph nodes, and C) mesenteric lymph nodes after culturing for 96 h in the presence or absence of OVA. In A and B, white bars represent RF group, black bars represent IP group and grey-striped bars represent FA group. In C, white bars summarize values without stimulus and striped bars represent values after OVA stimulation. Results are expressed as mean ± S.E.M. (n = 8). *p < 0.05 *vs*. non stimulated condition.

The percentage of TCRαβ cells, Tc and Th subsets, activated Th cells, B cells and B IgA^+^ subset from PP and MLN lymphocytes in the three studied groups is summarized in Fig 4. No differences between the groups were observed either in PP or MLN (Fig 4A and 4B), showing that both i.p. immunization and FA induction did not produce significant changes in the considered cell populations in either intestinal compartments.

After 96 h of OVA stimulation, the composition of MLN cells from RF animals did not significantly change (Fig 4C). Interestingly, in the IP group there was an increase in the TCRαβ cell proportion after OVA stimulation (*p* < 0.05). This increase corresponded to Tc and activated Th cells (*p* < 0.05). In cells from the FA group, no significant variations were observed in any of the studied MLN subsets after OVA stimulation.

### Cytokine production by spleen cells

The cytokine quantification of supernatants obtained from spleen cells isolated after two days of AR induction and cultured for four days with or without OVA was carried out. Those samples that had concentrations below the cutoff received a value corresponding to one-half the cutoff value, as previously described [55]. Spleen cells from RF animals did not produce detectable amounts of the studied cytokines (Table 1). After OVA stimulation, cells from the IP group increased their IL-2, IL-4 and IL-10 production with respect to that in the RF group (p < 0.05). On the contrary, the concentrations of cytokines from cells obtained from FA animals did not significantly differ from that of RF group, which could be due to the fact that IL-2 and IL-4 cytokines were only detected in 25% of FA animals, and IL-10 and IFN-γ in 50% and 75% of these animals, respectively. In comparison with the IP group, FA rats produced significantly lower amounts of IL-10 (*p* < 0.05).

**Table 1:** Cytokine production by spleen cells after stimulation with OVA.

Groups	IL-2 (pg/mL)	IL-4 (pg/mL)	IL-10 (pg/mL)	IFN-γ (pg/mL)
**Reference**	0.23	1.70	9.70	3.40
**Intraperitoneal**	54.18 ± 9.94*	104.34 ± 40.93*	803.3 ± 300.5*	26.94 ± 10.08
**Food allergy**	68.17 ± 25.32	38.97 ± 15.25	61.25 ± 33.54^ϕ^	7.55 ± 4.15

Results are expressed as mean ± S.E.M.

*p < 0.05 *vs*. RF group,

^ϕ^ *p* < 0.05 *vs*. IP group.

### Small intestine gene expression

**Fig 5:**
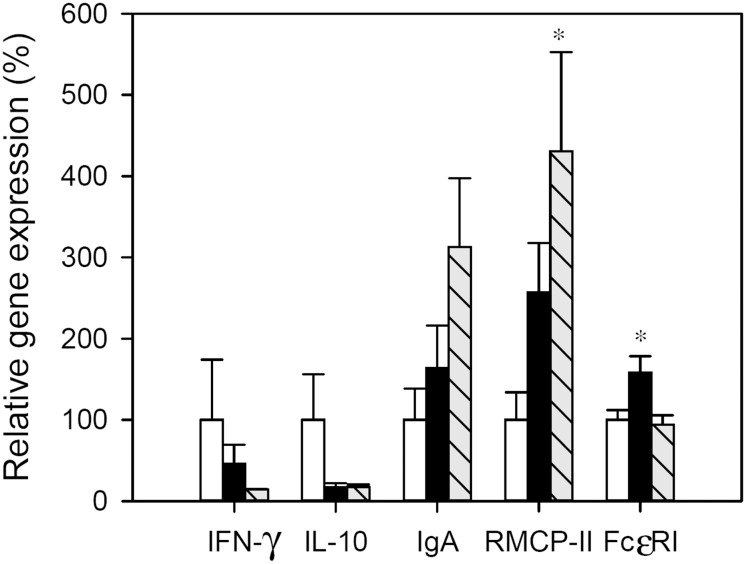
Relative gene expression in small intestine. Expression levels were normalized using HPRT as the endogenous housekeeping gene and were expressed as percentage in comparison with the RF group, which was considered as 100% gene expression. White bars represent RF group, black bars represent IP group and grey-striped bars represent FA group. Results are expressed as mean ± S.E.M. (n = 8). *p < 0.05 *vs*. RF group.

The gene expression of IFN-γ, IL-2, IL-4, IL-10, IgA, RMCP-II and FcεRI was analyzedin the SI at the end of the study (Fig 5). In both the IP and FA groups, IFN-γ and IL-10 gene expression was down-regulated whereas IgA mRNA levels increased but these changes did not achieve statistical significance. In the IP group a significant up-regulation of FcεRI gene expression was found in comparison with RF animals (*p* < 0.05) and RMCP-II mRNA levels also increased but not significantly. Regarding the FA group, the gene expression of RMCP-II increased about fourfold with respect to RF animals (*p* < 0.05), but no changes were detected in FcεRI. No significant amounts of mRNA of IL-2 and IL-4 were expressed in the small intestine wall from either the reference or immunized animals.

## Discussion

The present study provides the set-up and characterization of a FA model in BN rats, including the induction of an AR, carried out following the previous i.p. immunization with the allergen, OVA, together with alum and tBp and a subsequent daily oral administration of OVA for a period of three weeks. In order to establish specific biomarkers of FA, we compared the anti-allergen immune response and the AR obtained in this model with those achieved with only the i.p. immunization.

For the screening of drugs, nutritional interventions or immunotherapies to fight against allergies or for testing the allergenicity of new foods, many rat models of FA have been described, including those that only use the oral route [30–32], those that only use the i.p. route without adjuvant [40,56] and those that combine i.p. and oral administration [42]. Although BN rats are high IgE responders, similar to atopic humans, experimental procedures in this rat strain to induce oral sensitization without adjuvant are time-consuming and are not always able to generate a reproducible and effective FA model [20,30,37–39]. In fact, we previously tested a model in BN rats administered only by oral route and the result was that a few animals were sensitized and none produced specific IgE [39]. In contrast, other studies using several i.p. immunization protocols, with or without adjuvant, reported a successful production of specific IgE [40,56,57]. It is for this reason that we applied here an i.p. immunization with alum and tBp previous to the oral allergen administration.

As described in previous studies [54], the i.p. immunization of BN rats with OVA, alum and tBp induces the synthesis of specific antibodies in 100% of the animals, especially those isotypes related to Th2 immune response in rat, such as IgE, IgG1 and IgG2a [18,36,58]. The anti-OVA antibody profile, including specific IgE, is not surprising and can be attributed to both alum adjuvant and tBp which favor IgE synthesis [59,60]. Interestingly, when two weeks later a daily OVA solution was given orally, the specific antibody response was strengthened. This pattern was observed for serum IgG isotypes, which rose steeply during the first week of oral gavage, demonstrating that anti-OVA immune response was rapidly boosted by oral OVA administration. Similarly, OVA-specific IgE antibodies increased nearly twofold after one week of oral gavage but, however, when longer oral OVA administration was carried out, IgE serum concentrations decreased, following the same pattern as those that only received i.p. immunization. These results regarding serum anti-OVA antibody kinetics agree with those reported by Golias et al. [61] in a mouse model of FA obtained by two i.p. immunizations (two weeks apart) and oral feeding 14 days later every two days. In particular, this last study found that specific IgE response was already present before oral OVA administration, peaked during the first week after oral gavage and decreased later. Therefore, from the overall results concerning specific IgE, it could be suggested that only the first doses of the allergen administered are responsible for an exacerbation of the IgE synthesis and this response is lost with time. Overall, from the results concerning anti-OVA antibodies, it could be concluded that an effective FA model had been achieved because it produced the synthesis of specific antibodies in 100% of the animals and was relatively rapid since the highest specific IgE and IgG levels were reached one week after oral allergen administration, which was sooner than other reported models [34,37].

The FA model proposed here produced the synthesis of serum and intestinal anti-OVA IgA antibodies, which were not found when only i.p. immunization was carried out, thus demonstrating the stimulation of gut-associated lymphoid tissue. Although intestinal IgA is thought to contribute to gut homeostasis by limiting the uptake of oral antigens and it has been considered to have a protective role against oral sensitization [62], its role in food allergy is still controversial. In human FA, it has been reported that specific IgA2 levels (isotype mainly found in mucosa surfaces such as those of the intestine) increased when children became tolerant [63]. However, other authors reported that increased specific IgA was associated with a later FA [64] and that serum allergen-specific IgA seems not to be associated with food tolerance [65]. From our results, although oral challenge was performed with a high dose of oral OVA, the protective effect of intestinal IgA antibodies in the FA group was not observed because the measurement of AR provided similar results in both the FA and IP groups.

After AR induction, the FA model was characterized by a high increase in serum RMCP-II concentration, which again might reflect the stimulation of gut-associated lymphoid tissue because this protease is typical of activated mucosal mast cells [66]. In addition, other mediators released from mast cells produce vasodilatation and are responsible for the decrease in body temperature [67,68]. Animals immunized with only OVA by i.p. route and those immunized by i.p. route and subsequent oral OVA administration underwent a similar drop in body temperature after AR induction. There was no correlation between body temperature and the serum RMCP-II concentration, suggesting that other mast cells different from those in the intestinal mucosa could contribute to AR-induced hypothermia. On the other hand, AR caused an increase in intestinal permeability in both IP and FA groups, which must reflect the disrupted intestinal barrier after OVA immunization. It has been demonstrated that repeated OVA oral gavage produces an accumulation of RMCP-II in the intestine leading to altered motor responses in both the small intestine and the colon [69,70]. Nevertheless, it has been reported that an i.p. immunization produced a higher increase in intestinal permeability than an oral sensitization without an adjuvant, and this was attributed to the release of RMCP-II, among other mediators, which could increase the absorption by paracellular route [31]. From the results obtained here, rats with FA (i.p. and oral sensitization) seem to absorb βLG faster than the IP group because serum protein concentration tended to be higher at 30 min after βLG oral administration (60 min after AR induction) and disappeared faster. The collection of samples earlier than 30 min should confirm this suggestion and can shed some light as to whether there is any difference in intestinal permeability when OVA is given orally after the i.p. immunization. AR-induced behavioral changes were quantified by the decrease in motor activity as performed in a previous study [45], instead of using the classical score systems which require the subjective validation by the investigator [71,72]. The results after AR induction revealed a clear decrease of movements in comparison with the basal ones. However, when comparing the motor activity between the IP and FA groups, it could be observed that the decrease in motor activity induced by AR was similar in both groups. Therefore, from the results obtained after AR induction, it could be concluded that only the serum concentrations of RMCP-II, which were highly increased by oral OVA, clearly indicated the development of an FA model. Further studies on intestinal permeability should be directed to elucidate changes induced by oral allergen administration in this FA model. However, the decrease in body temperature and also in motor activity did not differ between IP and FA rats, which could be attributed to the similar serum IgE levels present at the end of the study.

Tissue samples obtained two days after AR induction allowed the detailed characterization of the FA process in comparison with the i.p. immunization. The study of lymphocyte composition in PP and MLN shows that neither the i.p. immunization nor the oral OVA administration changed the proportion of the main lymphocyte subsets in these intestinal compartments, at least at the moment when these samples were collected. These results did not agree with those of Ogawa et al. [43], which reported the accumulation of T lymphocytes in PP in a model of FA. Further studies carried out at different times could help to clarify this controversy, but from our results, it could be suggested that the characterization of lymphocyte phenotype in PP and MLN did not constitute a biomarker of FA induction. On the other hand, we observed that the proportion of T cells increased when MLN lymphocytes isolated from the IP group were specifically stimulated *in vitro*, but these results were not found in the FA group. These data could suggest the lymphocyte responsiveness in the IP group in contrast to the lymphocyte unresponsiveness after oral gavage of OVA for three weeks. This suggestion agrees with the cytokine results obtained from OVA-stimulated spleen cells, which show that only in the IP group was the amount of IL-4 and IL-10 released from spleen cells higher than that observed in the RF group, whereas the concentration of cytokines released by the FA group did not differ from reference values. In this sense, although some authors describe an increase of IL-4 and IL-10 in supernatants of spleen cultures of FA animals [34,73,74], other authors do not [42], and none of them compare the changes between i.p. immunization alone and i.p. together with an oral allergen administration. From these results it could be suggested that cytokines released from spleen cells collected after three weeks of allergen gavage did not reflect the oral sensitization process present in FA. Studies carried out in a previous phase of FA induction could better represent this response. In addition, other conditions of spleen cell incubation, such as a shorter stimulation and higher stimulus concentration, among others, could be better conditions for releasing representative cytokines. Nevertheless, it could be speculated that, at the end of the study, the continuous oral OVA administration produced a certain tolerance. This lack of response would not be reflected in the great synthesis of antibodies that occurred throughout the process, but would be only observed in the specific stimulation of cells collected at the end of the study.

Finally, the study of gene expression on intestinal tissue could reflect changes induced locally by oral OVA administration. We found that the gene expression of RMCP-II was significantly increased in FA animals, and these results agree with serum concentrations of this mediator and also with changes reported concerning the gene expression of this molecule in mice and rats with food allergies [33,35,43]. However, surprisingly the gene expression of FcεRI did not change with FA induction, although it did after i.p. immunization alone. It has been reported in mouse mast cells that the internalization of FcεRI is a mechanism of antigen-specific desensitization [75]. Therefore, the comparison of the results obtained in the FcεRI gene expression in IP and FA groups could endorse the idea that the FA group developed a certain tolerance from the continuous oral allergen administration.

In conclusion, by means of the combination of i.p. immunization followed by the oral gavage of the food allergen, we have established a rat model of FA that is effective because it was able to induce the synthesis of specific Th2-related antibodies, especially IgE, and consequently an AR after oral challenge in all animals. This fact represents a great advantage with respect to FA models only induced by oral route, which did not provide effective and reproducible results in all experiments. In addition, the allergic response development is faster than in other FA models described because one week after the oral administration of allergen i.e., three weeks after i.p. immunization, high levels of specific IgE were produced. In comparison with only i.p. immunization, the developed model provides much higher levels of specific IgG antibodies, achieving high amounts of Th2-related antibodies in rat (IgG1 and IgG2a), and also anti-OVA IgE, although the anaphylactic response after five weeks was similar in both groups. In addition, the levels of RMCP-II released after the anaphylaxis induction and the intestinal gene expression of this protease with respect to those of the i.p. immunization are the best biomarkers of the FA process. The results from *in vitro* antigen-specific activation of lymphocytes from spleen and mesenteric lymph nodes suggest a certain unresponsiveness state of these cells possibly induced by repeated oral doses of the allergen. Nevertheless, although further studies must confirm this hypothesis, the specific antibody response kinetics suggest that the best FA model could be obtained after only a week of oral OVA administration.

## References

[1] Boyce JA, Assa’ad A, Burks AW, Jones SM, Sampson HA, Wood RA, et al Guidelines for the diagnosis and management of food allergy in the United States: summary of the NIAID-Sponsored Expert Panel report. J Am Acad Dermatol 2011;64:175–192. 10.1016/j.jaad.2010.11.020 21167411

[2] Fox M, Mugford M, Voordouw J, Cornelisse-Vermaat J, Antonides G, de la Hoz Caballer B, et al Health sector costs of self-reported food allergy in Europe: a patient-based cost of illness study. Eur J Public Health 2013;23:757–762. 10.1093/eurpub/ckt010 23402805

[3] Patel D, Holdford D, Edwards E, Carroll N V. Estimating the economic burden of food-induced allergic reactions and anaphylaxis in the United States. J Allergy Clin Immunol 2011;128:110–115. 10.1016/j.jaci.2011.03.013 21489610

[4] Burks WA, Tang M, Sicherer S, Muraro A, Eigenmann PA, Ebisawa M, et al ICON: food allergy. J Allergy Clin Immunol 2012;129:906–920. 10.1016/j.jaci.2012.02.001 22365653

[5] Nwaru BI, Hickstein L, Panesar SS, Roberts G, Muraro A, Sheikh A, et al Prevalence of common food allergies in Europe: a systematic review and meta-analysis. Allergy 2014;69:992–1007. 10.1111/all.12423 24816523

[6] Sicherer SH, Sampson HA. Food allergy. J Allergy Clin Immunol 2006;117:S470–475. 16455349 10.1016/j.jaci.2005.05.048

[7] Sicherer SH, Sampson HA. Food allergy: Epidemiology, pathogenesis, diagnosis, and treatment. J Allergy Clin Immunol 2014;133:291–307; quiz 308. 10.1016/j.jaci.2013.11.020 24388012

[8] Ruiter B, Shreffler WG. The role of dendritic cells in food allergy. J Allergy Clin Immunol 2012;129:921–928. 10.1016/j.jaci.2012.01.080 22464669

[9] Blikslager AT, Moeser AJ, Gookin JL, Jones SL, Odle J. Restoration of barrier function in injured intestinal mucosa. Physiol Rev 2007;87:545–564. 17429041 10.1152/physrev.00012.2006

[10] Pabst O, Mowat AM. Oral tolerance to food protein. Mucosal Immunol 2012;5:232–239. 10.1038/mi.2012.4 22318493 PMC3328017

[11] Oyoshi MK, Oettgen HC, Chatila TA, Geha RS, Bryce PJ. Food allergy: Insights into etiology, prevention, and treatment provided by murine models. J Allergy Clin Immunol 2014;133:309–317. 10.1016/j.jaci.2013.12.1045 24636470 PMC3959655

[12] Buchanan BB, Frick OL. The dog as a model for food allergy. Ann N Y Acad Sci 2002;964:173–183. 12023204 10.1111/j.1749-6632.2002.tb04142.x

[13] Helm RM, Furuta GT, Stanley JS, Ye J, Cockrell G, Connaughton C, et al A neonatal swine model for peanut allergy. J Allergy Clin Immunol 2002;109:136–142. 11799380 10.1067/mai.2002.120551

[14] Teuber SS, del Val G, Morigasaki S, Jung HR, Eisele PH, Frick OL, et al The atopic dog as a model of peanut and tree nut food allergy. J Allergy Clin Immunol 2002;110:921–927. 12464960 10.1067/mai.2002.130056

[15] Piacentini GL, Vicentini L, Bodini A, Mazzi P, Peroni DG, Maffeis C, et al Allergenicity of a hydrolyzed rice infant formula in a guinea pig model. Ann Allergy Asthma Immunol 2003;91:61–64. 12877451 10.1016/S1081-1206(10)62060-1

[16] Ganeshan K, Neilsen C V, Hadsaitong A, Schleimer RP, Luo X, Bryce PJ. Impairing oral tolerance promotes allergy and anaphylaxis: a new murine food allergy model. J Allergy Clin Immunol 2009;123:231–238. 10.1016/j.jaci.2008.10.011 19022495 PMC2787105

[17] Vinje NE, Larsen S, Løvik M. A mouse model of lupin allergy. Clin Exp Allergy 2009;39:1255–1266. 10.1111/j.1365-2222.2009.03269.x 19438583

[18] Sun N, Zhou C, Pu Q, Wang J, Huang K, Che H. Allergic reactions compared between BN and Wistar rats after oral exposure to ovalbumin. J Immunotoxicol 2013;10:67–74. 10.3109/1547691X.2012.693546 23110332

[19] Ahuja V, Quatchadze M, Ahuja V, Stelter D, Albrecht A, Stahlmann R. Evaluation of biotechnology-derived novel proteins for the risk of food-allergic potential: advances in the development of animal models and future challenges. Arch Toxicol 2010;84:909–917. 10.1007/s00204-010-0582-0 20842347

[20] Kimber I, Dearman RJ, Penninks AH, Knippels LMJ, Buchanan RB, Hammerberg B, et al Assessment of protein allergenicity on the basis of immune reactivity: animals models. Environ Health Perspect 2002;111:1125–1130. 10.1289/ehp.5813 PMC1241562 12826485

[21] Kitagawa S, Zhang S, Harari Y, Castro GA. Relative allergenicity of cow’s milk and cow's milk-based formulas in an animal model. Am J Med Sci 1995;310:183–187. 7485221 10.1097/00000441-199511000-00002

[22] Fritsché R. Animal models in food allergy: assessment of allergenicity and preventive activity of infant formulas. Toxicol Lett 2003;140–141:303–309. 10.1016/s0378-4274(03)00026-2 12676478

[23] Ando T, Matsumoto K, Namiranian S, Yamashita H, Glatthorn H, Kimura M, et al Mast cells are required for full expression of allergen/SEB-induced skin inflammation. J Invest Dermatol 2013;133:2695–2705. 10.1038/jid.2013.250 23752044 PMC3830701

[24] Fattouh R, Pouladi MA, Alvarez D, Johnson JR, Walker TD, Goncharova S, et al House dust mite facilitates ovalbumin-specific allergic sensitization and airway inflammation. Am J Respir Crit Care Med 2005;172:314–321. 15879422 10.1164/rccm.200502-198OC

[25] Li X-M, Schofield BH, Huang C, Kleiner GI, Sampson HA. A murine model of IgE-mediated cow’s milk hypersensitivity. J Allergy Clin Immunol 1999;103:206–214. 9949309 10.1016/s0091-6749(99)70492-6

[26] Bailón E, Cueto-Sola M, Utrilla P, Rodríguez-Ruiz J, Garrido-Mesa N, Zarzuelo A, et al A shorter and more specific oral sensitization-based experimental model of food allergy in mice. J Immunol Methods 2012;381:41–49. 10.1016/j.jim.2012.04.007 22542400

[27] Sun J, Arias K, Alvarez D, Fattouh R, Walker T, Goncharova S, et al Impact of CD40 ligand, B cells, and mast cells in peanut-induced anaphylactic responses. J Immunol 2007;179:6696–6703. 17982059 10.4049/jimmunol.179.10.6696

[28] Aldemir H, Bars R, Herouet-Guicheney C. Murine models for evaluating the allergenicity of novel proteins and foods. Regul Toxicol Pharmacol 2009;54:S52–57. 10.1016/j.yrtph.2008.11.004 19100305

[29] Penninks AH, Knippels LM. Determination of protein allergenicity: studies in rats. Toxicol Lett 2001;120:171–180. 11323175 10.1016/s0378-4274(01)00275-2

[30] Knippels LM, Penninks AH, van Meeteren M, Houben GF. Humoral and cellular immune responses in different rat strains on oral exposure to ovalbumin. Food Chem Toxicol 1999;37:881–888. 10506012 10.1016/s0278-6915(99)00064-2

[31] Knippels LMJ, Penninks AH, Smit JJ, Houben GF. Immune-mediated effects upon oral challenge of ovalbumin-sensitized Brown Norway rats: further characterization of a rat food allergy model. Toxicol Appl Pharmacol 1999;156:161–169. 10222308 10.1006/taap.1999.8641

[32] Knippels LM, Penninks AH, Houben GF. Continued expression of anti-soy protein antibodies in rats bred on a soy protein-free diet for one generation: the importance of dietary control in oral sensitization research. J Allergy Clin Immunol 1998;101:815–820. 9648709 10.1016/S0091-6749(98)70309-4

[33] Zhong Y, Huang J, Tang W, Chen B, Cai W. Effects of probiotics, probiotic DNA and the CpG oligodeoxynucleotides on ovalbumin-sensitized Brown-Norway rats via TLR9/NF-κB pathway. FEMS Immunol Med Microbiol 2012;66:71–82. 10.1111/j.1574-695X.2012.00991.x 22612777

[34] Huang J, Zhong Y, Cai W, Zhang H, Tang W, Chen B. The effects of probiotics supplementation timing on an ovalbumin-sensitized rat model. FEMS Immunol Med Microbiol 2010;60:132–141. 10.1111/j.1574-695X.2010.00727.x 20846358

[35] Cao S, He X, Xu W, Luo Y, Ran W, Liang L, et al Potential allergenicity research of Cry1C protein from genetically modified rice. Regul Toxicol Pharmacol 2012;63:181–187. 10.1016/j.yrtph.2012.03.017 22504668

[36] De Jonge JD, Baken KA, Konings J, Pennings JL, Ezendam J, Van Loveren H. Gene expression changes in the mesenteric lymph nodes of rats after oral peanut extract exposure. J Immunotoxicol 2008;5:385–394. 10.1080/15476910802586126 19404872

[37] Pilegaard K, Madsen C. An oral Brown Norway rat model for food allergy: comparison of age, sex, dosing volume, and allergen preparation. Toxicology 2004;196:247–257. 15036751 10.1016/j.tox.2003.11.010

[38] Dearman RJ, Caddick H, Stone S, Basketter DA, Kimber I. Characterization of antibody responses induced in rodents by exposure to food proteins: influence of route of exposure. Toxicology 2001;167:217–231. 11578801 10.1016/s0300-483x(01)00462-0

[39] Camps-Bossacoma M, Abril-Gil M, Franch À, Pérez-Cano FJ, Castell M. Induction of a model of oral sensitization in rat. Clin Immunol Endocr Metab Drugs 2015; in press.

[40] Bøgh KL, Kroghsbo S, Dahl L, Rigby NM, Barkholt V, Mills ENC, et al Digested Ara h 1 has sensitizing capacity in Brown Norway rats. Clin Exp Allergy 2009;39:1611–1621. 10.1111/j.1365-2222.2009.03333.x 19689460

[41] Kroghsbo S, Bøgh KL, Rigby NM, Mills ENC, Rogers A, Madsen CB. Sensitization with 7S globulins from peanut, hazelnut, soy or pea induces IgE with different biological activities which are modified by soy tolerance. Int Arch Allergy Immunol 2011;155:212–224. 10.1159/000321200 21282960

[42] Ahrens B, Quarcoo D, Buhner S, Reese G, Vieths S, Hamelmann E. Development of an animal model to evaluate the allergenicity of food allergens. Int Arch Allergy Immunol 2014;164:89–96. 10.1159/000363109 24903216

[43] Ogawa T, Miura S, Tsuzuki Y, Ogino T, Teramoto K, Inamura T, et al Chronic allergy to dietary ovalbumin induces lymphocyte migration to rat small intestinal mucosa that is inhibited by MAdCAM-1. Am J Physiol Gastrointest Liver Physiol 2004;286:G702–710. 14670821 10.1152/ajpgi.00183.2003

[44] Dong W, Selgrade MK, Gilmour MI. Systemic administration of Bordetella pertussis enhances pulmonary sensitization to house dust mite in juvenile rats. Toxicol Sci 2003;72:113–121. 12604840 10.1093/toxsci/kfg015

[45] Abril-Gil M, Garcia-Just A, Cambras T, Pérez-Cano FJ, Cristina C, Franch À, et al Motor activity as unbiased variable to assess anaphylactic shock in allergic rats. Exp Biol Med (Maywood) 2015; 1535370215573393, first published on February 25.

[46] Chiesa JJ, Cambras T, Carpentieri AR, Díez-Noguera A. Arrhythmic rats after SCN lesions and constant light differ in short time scale regulation of locomotor activity. J Biol Rhythms 2010;25:37–46. 10.1177/0748730409352843 20075299

[47] Albert N, da Silva C, Díez-Noguera A, Cambras T. Different adaptation of the motor activity rhythm to chronic phase shifts between adolescent and adult rats. Behav Brain Res 2013;252:347–355. 10.1016/j.bbr.2013.06.025 23792134

[48] Ramiro-Puig E, Pérez-Cano FJ, Ramos-Romero S, Pérez-Berezo T, Castellote C, Permanyer J, et al Intestinal immune system of young rats influenced by cocoa-enriched diet. J Nutr Biochem 2008;19:555–565. 18061430 10.1016/j.jnutbio.2007.07.002

[49] Pérez-Berezo T, Franch A, Ramos-Romero S, Castellote C, Pérez-Cano FJ, Castell M. Cocoa-enriched diets modulate intestinal and systemic humoral immune response in young adult rats. Mol Nutr Food Res 2011;55:S56–66. 10.1002/mnfr.201000588 21462334

[50] Pérez-Berezo T, Ramírez-Santana C, Franch A, Ramos-Romero S, Castellote C, Pérez-Cano FJ, et al Effects of a cocoa diet on an intestinal inflammation model in rats. Exp Biol Med 2012;237:1181–1188. 10.1258/ebm.2012.012083 23104506

[51] Sakamoto Y, Ohtsuka T, Yoshida H, Ohto K, Onobori M, Matsumoto T, et al Time course of changes in the intestinal permeability of food-sensitized rats after oral allergen challenge. Pediatr Allergy Immunol 1998;9:20–24. 9560838 10.1111/j.1399-3038.1998.tb00295.x

[52] Massot-Cladera M, Pérez-Berezo T, Franch A, Castell M, Pérez-Cano FJ. Cocoa modulatory effect on rat faecal microbiota and colonic crosstalk. Arch Biochem Biophys 2012;527:105–112. 10.1016/j.abb.2012.05.015 22663919

[53] Livak KJ, Schmittgen TD. Analysis of relative gene expression data using real-time quantitative PCR and the 2(-Delta Delta C(T)) Method. Methods 2001;25:402–408. 11846609 10.1006/meth.2001.1262

[54] Abril-Gil M, Massot-Cladera M, Pérez-Cano FJ, Castellote C, Franch À, Castell M. A diet enriched with cocoa prevents IgE synthesis in a rat allergy model. Pharmacol Res 2012;65:603–608. 10.1016/j.phrs.2012.02.001 22342543

[55] Zanardo V, Golin R, Amato M, Trevisanuto D, Favaro F, Faggian D, et al Cytokines in human colostrum and neonatal jaundice. Pediatr Res 2007;62:191–194. 17597660 10.1203/PDR.0b013e31809871c9

[56] Bøgh KL, Barkholt V, Madsen CB. The sensitising capacity of intact β-lactoglobulin is reduced by co-administration with digested β-lactoglobulin. Int Arch Allergy Immunol 2013;161:21–36. 10.1159/000351238 23257607

[57] Bellou A, Saint-Laudy J, Knippels L, Montémont C, Vauthier E, Gerard P, et al Brown Norway rat ovalbumin-specific immunoglobulin E antibodies increase the human basophil expression of CD63 marker. Scand J Immunol 2003;57:271–278. 12641656 10.1046/j.1365-3083.2003.01233.x

[58] Gracie JA, Bradley JA. Interleukin-12 induces interferon-γ-dependent switching of IgG alloantibody subclass. Eur J Immunol 1996;8:1217–1221. 10.1002/eji.1830260605 8647195

[59] Terhune TD, Deth RC. How aluminum adjuvants could promote and enhance non-target IgE synthesis in a genetically-vulnerable sub-population. J Immunotoxicol 2013;10:210–222. 10.3109/1547691X.2012.708366 22967010

[60] Saavedra Y, Vergara P. Hypersensitivity to ovalbumin induces chronic intestinal dysmotility and increases the number of intestinal mast cells. Neurogastroenterol Motil 2005;17:112–122. 15670271 10.1111/j.1365-2982.2004.00597.x

[61] Golias J, Schwarzer M, Wallner M, Kverka M, Kozakova H, Srutkova D, et al Heat-induced structural changes affect OVA-antigen processing and reduce allergic response in mouse model of food allergy. PLoS One 2012;7: e37156 10.1371/journal.pone.0037156 22629361 PMC3357411

[62] Berin MC, Mayer L. Immunophysiology of experimental food allergy. Mucosal Immunol 2009;2:24–32. 10.1038/mi.2008.72 19079331

[63] Konstantinou GN, Nowak-Węgrzyn A, Bencharitiwong R, Bardina L, Sicherer SH, Sampson HA. Egg-white-specific IgA and IgA2 antibodies in egg-allergic children: is there a role in tolerance induction? Pediatr Allergy Immunol 2014;25:64–70. 10.1111/pai.12143 24118158 PMC4134474

[64] Orivuori L, Mustonen K, Roduit C, Braun-Fahrländer C, Dalphin J-C, Genuneit J, et al Immunoglobulin A and immunoglobulin G antibodies against β-lactoglobulin and gliadin at age 1 associate with immunoglobulin E sensitization at age 6. Pediatr Allergy Immunol 2014;25:329–337. 10.1111/pai.12246 24953294

[65] Vazquez-Ortiz M, Pascal M, Juan M, Alsina L, Martín-Mateos MA, Plaza AM. Serum allergen-specific IgA is not associated with natural or induced tolerance to egg in children. Allergy 2013;68:1327–1332. 10.1111/all.12217 24004369

[66] Gibson S, Mackeller A, Newlands G, Miller H. Phenotypic expression of mast cell granule proteinases. Distribution of mast cell proteinases I and II in the rat digestive system. Immunology 1987;62:621–627. 3323033 PMC1454143

[67] Makabe-Kobayashi Y, Hori Y, Adachi T, Ishigaki-Suzuki S, Kikuchi Y, Kagaya Y, et al The control effect of histamine on body temperature and respiratory function in IgE-dependent systemic anaphylaxis. J Allergy Clin Immunol 2002;110:298–303. 12170272 10.1067/mai.2002.125977

[68] Khodoun M, Strait R, Orekov T, Hogan S, Karasuyama H, Herbert DR, et al Peanuts can contribute to anaphylactic shock by activating complement. J Allergy Clin Immunol 2009;123:342–351. 10.1016/j.jaci.2008.11.004 19121857 PMC2670761

[69] Traver E, Torres R, de Mora F, Vergara P. Mucosal mast cells mediate motor response induced by chronic oral exposure to ovalbumin in the rat gastrointestinal tract. Neurogastroenterol Motil 2010;22:e34–43. 10.1111/j.1365-2982.2009.01377.x 19682267

[70] Jardí F, Martínez V, Vergara P. NGF is involved in oral ovalbumin-induced altered colonic contractility in rats: evidence from the blockade of TrkA receptors with K252a. Neurogastroenterol Motil 2012;24:e580–590. 10.1111/nmo.12027 23072452

[71] Leonard SA, Martos G, Wang W, Nowak-Węgrzyn A, Berin MC. Oral immunotherapy induces local protective mechanisms in the gastrointestinal mucosa. J Allergy Clin Immunol 2012;129:1579–1587. 10.1016/j.jaci.2012.04.009 22554705 PMC3367084

[72] Shindo T, Kanazawa Y, Saito Y, Kojima K, Ohsawa M, Teshima R. Effective induction of oral anaphylaxis to ovalbumin in mice sensitized by feeding of the antigen with aid of oil emulsion and salicylate. J Toxicol Sci 2012;37:307–315. 22467021 10.2131/jts.37.307

[73] Duncker SC, Philippe D, Martin-Paschoud C, Moser M, Mercenier A, Nutten S. *Nigella sativa* (black cumin) seed extract alleviates symptoms of allergic diarrhea in mice, involving opioid receptors. PLoS One 2012;7:e39841 10.1371/journal.pone.0039841 22768141 PMC3387213

[74] Okada Y, Oh-oka K, Nakamura Y, Ishimaru K, Matsuoka S, Okumura K, et al Dietary resveratrol prevents the development of food allergy in mice. PLoS One 2012;7:e44338 10.1371/journal.pone.0044338 22962611 PMC3433457

[75] Oka T, Rios EJ, Tsai M, Kalesnikoff J, Galli SJ. Rapid desensitization induces internalization of antigen-specific IgE on mouse mast cells. J Allergy Clin Immunol 2013;132:922–32. 10.1016/j.jaci.2013.05.004 23810240 PMC3789647

